# A global model of avian influenza prediction in wild birds: the importance of northern regions

**DOI:** 10.1186/1297-9716-44-42

**Published:** 2013-06-13

**Authors:** Keiko A Herrick, Falk Huettmann, Michael A Lindgren

**Affiliations:** 1 Ecological Wildlife Habitat Analysis of the Land- and Seascape (EWHALE) Lab, Biology and Wildlife Department, University of Alaska Fairbanks, Fairbanks, AK 99775, USA; 2 Institute of Arctic Biology, University of Alaska Fairbanks, Fairbanks, AK 99775-7000, USA; 3 Scenarios Network for Alaska and Arctic Planning, University of Alaska Fairbanks, Fairbanks, AK 99775, USA

## Abstract

Avian influenza virus (AIV) is enzootic to wild birds, which are its natural reservoir. The virus exhibits a large degree of genetic diversity and most of the isolated strains are of low pathogenicity to poultry. Although AIV is nearly ubiquitous in wild bird populations, highly pathogenic H5N1 subtypes in poultry have been the focus of most modeling efforts. To better understand viral ecology of AIV, a predictive model should 1) include wild birds, 2) include all isolated subtypes, and 3) cover the host’s natural range, unbounded by artificial country borders. As of this writing, there are few large-scale predictive models of AIV in wild birds. We used the Random Forests algorithm, an ensemble data-mining machine-learning method, to develop a global-scale predictive map of AIV, identify important predictors, and describe the environmental niche of AIV in wild bird populations. The model has an accuracy of 0.79 and identified northern areas as having the highest relative predicted risk of outbreak. The primary niche was described as regions of low annual rainfall and low temperatures. This study is the first global-scale model of low-pathogenicity avian influenza in wild birds and underscores the importance of largely unstudied northern regions in the persistence of AIV.

## Introduction

The influenza viruses that caused the four deadliest human pandemics of the past century (1918, 1957, 1968, 2009) contained gene segments from avian influenza acquired through recent reassortment events (reviewed in [1]). Influenza is thought to have originated in wild birds, and waterfowl are considered the primary reservoir. Avian influenza virus (AIV) most commonly infects *Anseriformes*, *Passeriformes*, and *Charadriiformes* in wild populations, particularly family *Anatidae*[2]. The Asian strains of the highly pathogenic H5N1 AIV subtype in poultry have received the most attention because of economic losses caused by this subtype, the virus’s transmissibility from chicken to human [3,4], and fears over a new human influenza pandemic [5]. However, HPAI H5N1 is not the only strain with pandemic potential: cases of human infection with interspecies H7 and H9 subtypes have been reported [6,7] and others, such as H6, can be highly pathogenic to poultry. The vast majority of influenza strains are of low pathogenicity to poultry, but because AIV is a virus of great diversity with the potential for rapid evolution [8], the full range of its variation should be considered rather than just focusing on a single strain or subtype. A massive reservoir of genetic diversity for potential reassortment of AIV exists in wild bird populations, from which nearly all combinations of hemagglutinin (H) and neuraminidase (N) subtypes have been isolated [9-11].

A number of ongoing surveillance projects record the subtypes of AIV isolated from wild birds [9,10,12,13]. Large cooperative databases, such as the Influenza Research Database (IRD), curate the surveillance efforts of multiple institutions. IRD provides an opportunity to apply predictive modeling to AIV on a global scale. In the prediction and risk assessment of infectious diseases, geographic information systems (GIS) and predictive modeling techniques are important tools [14]. Predictive models of Chagas disease [15], malaria [16], leishmaniasis [17], and Lyme disease [18] have been used to map disease prevalence and identify important factors contributing to risk. Several models have assessed risk factors for H5N1 in domestic poultry and produced high resolution models for India [19], Vietnam [20], and China [21]. One predictive map of multiple species of wild birds and subtypes of AIV was developed for the continental United States [22], and one for flyways adjacent to the Pacific Rim [23].

As of this writing, there are no global-scale predictions of LPAI in wild birds. The development of a global model could be an important tool in the management and risk assessment of AIV in the interest of public and animal health. Our model extends the value of AIV surveillance efforts by using the data for predictive purposes, not simply for descriptive purposes. In addition, a global model encompasses the distribution range of important reservoir species, many of which travel vast distances [24] in cross-continental migratory journeys to and from breeding grounds each year. We used ensemble data-mining machine-learning methods to 1) identify important predictor variables, 2) quantitatively describe the environmental niche of AIV in wild bird populations, and 3) develop a near-global-scale predictive map (excluding Antarctica) of AIV based on this described niche.

## Materials and methods

### Wild bird data

Sample data points of AIV-negative and AIV-positive data for wild birds were obtained from the Influenza Research Database online [25]. This dataset spans five years (2005–2010) of surveillance data providing georeferenced collection coordinates for each sample, species name, AIV-positive or –negative status (determined by the collecting institution), viral subtype (where available), and many other collection specifics. We did not distinguish between high- and low-pathogenicity AIV strains in our dataset. We groomed the database to remove samples from domestic species and samples from unidentified species (listed as “Unknown”). In addition, this version of the database contained many instances where the latitude and longitude values were inverted; we examined each point for a match between GPS coordinates and collection location, and corrected it if the error was obvious or removed it if uncertainty remained. We randomly divided the data points into two pools for training the model (47 898 points) and testing the model (12 080 points) using MS Excel and imported both sets as point layers into ArcMap v.10.0 (Esri, Redlands USA).

### Environmental variable layers

Forty-one predictor variable layers for ArcGIS were acquired from open source projects and included bioclimatic, geographic, and anthropogenic variables (Table 1). The extent of this model is bounded by these data layers, which exclude Antarctica. Bioclimatic variables included mean temperature for each month, for quarters (e.g. wettest quarter), and annual means for precipitation and temperature. A number of time-dependent variables were included (i.e. mean temperature in January - December) and were manipulated in order to maintain their relevance to collection locations in the Southern Hemisphere. For points with negative latitude values, time-dependent variables were shifted by 6 months, such that months were correctly associated with the austral seasons. Geographic variables included elevation, which has been identified as an important factor in other AIV models [19], and lakes, rivers, and wetlands, which are important to waterfowl. We calculated some layers from existing predictor variables using the Spatial Analyst Tool in ArcMap. The distances from fresh water features and coastline were calculated using the Euclidean Distance Tool. Slope was calculated from elevation and aspect was in turn calculated from slope. Anthropogenic variables included indices of human manipulation, infrastructure, and population density. Due to the importance of chickens and pigs in the transmission of AIV to humans, we included predicted poultry and pig densities [26,27]. Not all layers included Antarctica, so the entire continent was excluded from the study area (layers trimmed at −57° latitude) to prevent biases in calculation. We then used the Geospatial Modeling Environment (GME; [28]) to intersect, or extract the values of the predictor variables at the same geographic coordinates as the sample data points. GME adds the values of each predictor variable to the database as an additional column. The intersected database is then imported into ArcMap for visualization. Layers and metadata are stored at and can be obtained from the Ecological Wildlife Habitat Analysis of the Land- and Seascape (EWHALE) Lab at the University of Alaska Fairbanks (UAF).

**Table 1:** The predictor variables used by the Random Forests algorithm to create a global prediction map for avian influenza virus in wild birds.

**Predictor variable**	**Details**	**VIS**	**Project source**
Annual precipitation (mm)	In mm; 30 arc-seconds, 1 km spatial resolution	100.0	WorldClim [29]
Mean temperature, June (°C)	In °C; 30 arc-seconds, 1 km spatial resolution	85.2	WorldClim
Mean temperature, April (°C)	In °C; 30 arc-seconds, 1 km spatial resolution	76.1	WorldClim
Precipitation of driest quarter (mm)	In mm; 30 arc-seconds, 1 km spatial resolution	68.4	WorldClim
Mean temperature, November (°C)	In °C; 30 arc-seconds, 1 km spatial resolution	64.9	WorldClim
Precipitation seasonality	In mm; 30 arc-seconds, 1 km spatial resolution	63.1	WorldClim
Mean temperature of driest quarter (°C)	In °C; 30 arc-seconds, 1 km spatial resolution	62.5	WorldClim
Annual mean temperature (°C)	In °C; 30 arc-seconds, 1 km spatial resolution	54.8	WorldClim
Mean temperature, February (°C)	In °C; 30 arc-seconds, 1 km spatial resolution	49.7	WorldClim
Mean temperature, January (°C)	In °C; 30 arc-seconds, 1 km spatial resolution	45.7	WorldClim
Temperature seasonality	Standard deviation × 100	45.0	WorldClim
Precipitation of wettest quarter (mm)	In mm; 30 arc-seconds, 1 km spatial resolution	42.9	WorldClim
Mean temperature, December (°C)	In °C; 30 arc-seconds, 1 km spatial resolution	38.6	WorldClim
Maximum temperature of warmest month (°C)	In °C; 30 arc-seconds, 1 km spatial resolution	38.0	WorldClim
Precipitation of driest month (mm)	In mm; 30 arc-seconds, 1 km spatial resolution	37.5	WorldClim
Mean temperature, October (°C)	In °C; 30 arc-seconds, 1 km spatial resolution	36.1	WorldClim
Mean temperature, September (°C)	In °C; 30 arc-seconds, 1 km spatial resolution	32.6	WorldClim
Precipitation of coldest quarter (mm)	In mm; 30 arc-seconds, 1 km spatial resolution	32.3	WorldClim
Population density (persons/km^2^)	Population density for 2010, 2.5’ resolution, persons/km^2^	29.3	Gridded Popn of the World, v.3 [30]
Mean temperature of coldest quarter (°C)	In °C; 30 arc-seconds, 1 km spatial resolution	28.4	WorldClim
Mean temperature, July (°C)	In °C; 30 arc-seconds, 1 km spatial resolution	28.3	WorldClim
Isothermality (°C)	(Mean Diurnal Range/Temperature Annual Range); In °C; 30 arc-seconds, 1 km spatial resolution	26.6	WorldClim
Mean temperature of wettest quarter (°C)	In °C; 30 arc-seconds, 1 km spatial resolution	25.2	WorldClim
Mean diurnal range (°C)	In °C, (mean of monthly temperature(max – min))	24.3	WorldClim
Mean temperature, August (°C)	In °C; 30 arc-seconds, 1 km spatial resolution	22.7	WorldClim
Mean temperature, March (°C)	In °C; 30 arc-seconds, 1 km spatial resolution	21.0	WorldClim
Temperature annual range (°C)	In °C; 30 arc-seconds, 1 km spatial resolution	18.4	WorldClim
Elevation (m)	In m; 30 arc-seconds, 1 km spatial resolution	18.3	WorldClim
Predicted pig density (per km^2^)	Animal density/km^2^; 3 min of arc	18.0	Gridded livestock of the world [31]
Mean temperature of warmest quarter (°C)	In °C; 30 arc-seconds, 1 km spatial resolution	17.0	WorldClim
Precipitation of wettest month (mm)	In mm; 30 arc-seconds, 1 km spatial resolution	15.0	WorldClim
Distance from coast (m)	Calculated from coastline	12.6	Esri
Precipitation of warmest quarter (mm)	In mm; 30 arc-seconds, 1 km spatial resolution	12.1	WorldClim
Minimum temperature of coldest month (°C)	In °C; 30 arc-seconds, 1 km spatial resolution	11.2	WorldClim
Human footprint index	Percentage of relative human influence (0–100)	10.4	Last of the Wild [32]
Distance from rivers, lakes, or wetlands	Calculated from combined large and small lake polygons, nd lakes and wetlands grid	9.7	Global Lakes and Wetlands Database [33]
Slope	Calculated from elevation	9.0	Esri
Human influence index	Summative index of human disturbance (0–72)	8.8	Last of the Wild [32]
Predicted poultry density (per km^2^)	Animal density/km^2^; 3 min of arc	7.9	Gridded livestock of the world [31]
Aspect	positive degrees from 0 to 359.9, measured clockwise from north; calculated from slope	6.7	Esri
Mean temperature, May (°C)	In °C; 30 arc-seconds, 1 km spatial resolution	5.4	WorldClim

Variables are listed in order of their Variable Importance Score (VIS) or relative contribution to model accuracy as calculated by Random Forests.

### Defining the outbreak niche

We used the Random Forests algorithm [34], an ensemble data-mining machine-learning method, to identify the variables that best predicted the AIV-positive niche. We chose this particular algorithm because it is a powerful method of data-mining that performs with equal or superior accuracy to other algorithms (such as TreeNet, MARS, and Regression Tree Analysis) when used in ecological prediction [35,36]. Random Forests is relatively immune to overfitting and noise [34], which is a valuable feature when many similar predictor variables are incorporated. In addition, Random Forests ranks predictor variables by their contribution to model accuracy and the Variable Importance Scores (VIS) are normalized to the highest scoring variable. Using the pool of training data, we ran the Random Forests analysis method for classification trees in Salford Predictive Miner (Salford Systems, San Diego USA) with the following settings: class weights were balanced to up-weight the smaller number of AIV-positive samples against AIV-negative samples; the number of trees was set to 500; and seven predictors were used at each node [34].

The top five variables with the highest VIS were chosen for further examination. To compare the number of AIV-positive and -negative samples taken across each variable’s range of values we plotted density using Spotfire S+ (TIBCO, v.8.2, Palo Alto USA). The ranges within which peaks occur suggest underlying mechanisms, which may be driving AIV outbreaks. Partial dependence plots were produced using the “partialPlot()” command in the RandomForest package [37] in R statistical programming language [38]. Partial dependence can be thought of as an index summarizing the quantified relationship of a predictor with the response variable after averaging the noise of non-relevant predictors [39]. Partial dependence plots can be useful in illustrating general trends in model accuracy’s dependence on predictors. The partial dependence of a variable’s effect is best understood by examining general patterns in relation to the values of the predictor variable rather than the specific values of partial dependence.

**Figure 1:**
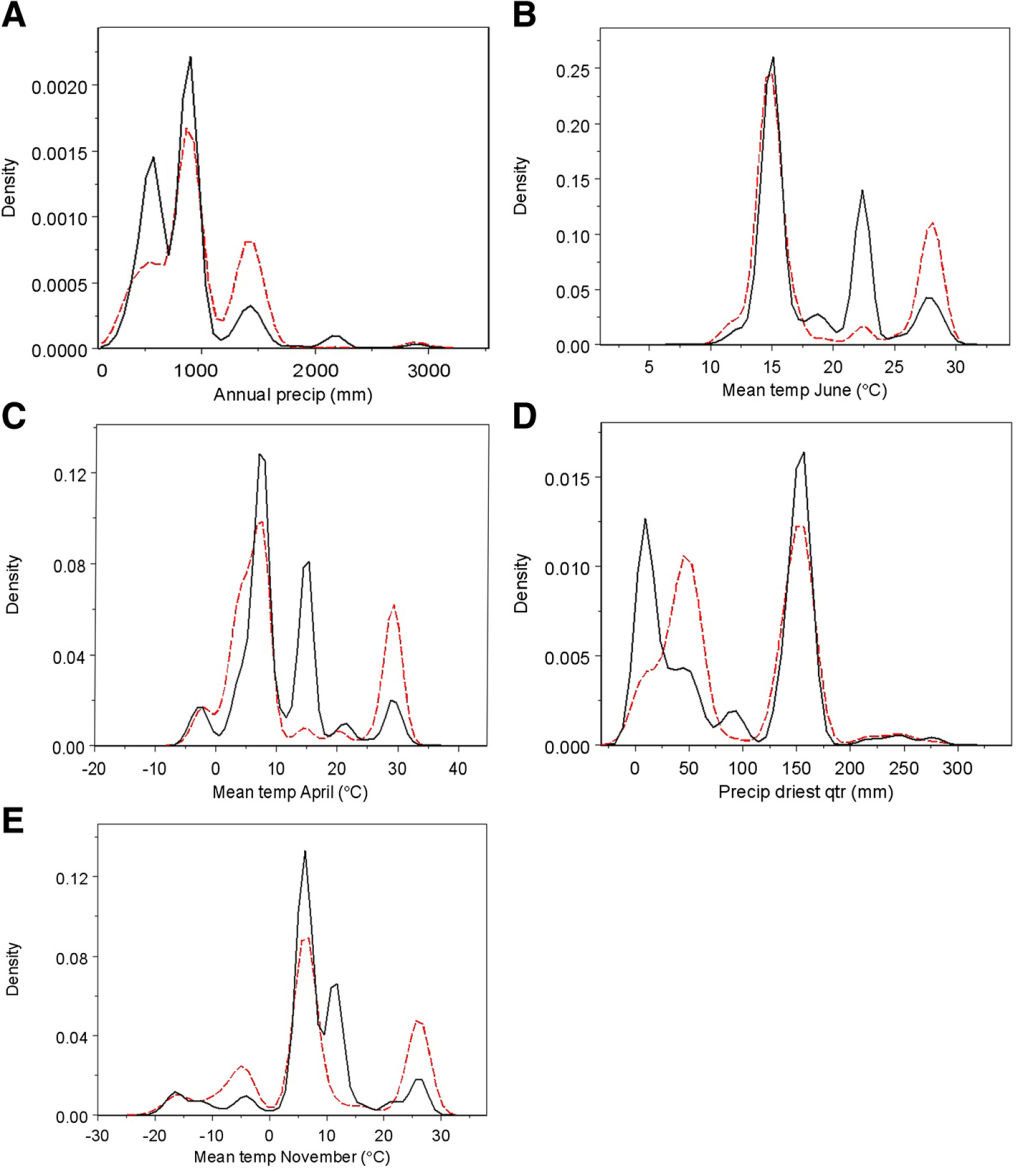
**Density plots for important variables.** Density plots for the variables with the five highest variable importance scores as calculated by Random Forests in the accuracy of the predictive model of avian influenza in wild birds (**A**-**E**). The density, or the likelihood of a variable to take on a value, of AIV-positive samples for each variable is represented by red dotted lines, the AIV-negative by black solid lines.

As a negative control, we calculated AUC for the AIV-positive status of the training subset against three individual predictors: the predictor with the highest importance score, the lowest importance score, and Annual Mean Temperature. We examined partial dependence plots for the general relationship between the range of predictor values and AIV-positivity (e.g. Figure 1a for Annual Precipitation). Predictor values for each point in the subset of training data were normalized between 0 and 1. If the relationship was negative, then the values were inverted such that low values of the variable predicted high occurrence. All three sets of normalized values were then subjected to AUC calculations.

### Predictive map

To predict the relative occurrence of AIV in unsampled areas, we applied the model to a lattice of points spaced 100 km apart and calculated a predicted value for each point. Random Forests expresses the predicted occurrence of AIV as a Relative Occurrence Index (ROI) rather than a probability score [40]. In ArcMap, we applied the Inverse Distance Weighted Tool (IDW) to interpolate these ROI values between the points, and generated a map of predicted AIV outbreak locations. The final map was projected as Robinson (sphere) with the central meridian at 145° so that Africa and Europe are displayed intact.

To evaluate the performance of the model, we calculated the Receiver Operating Characteristic (ROC) curve by plotting true positive points (AIV-positive status) against false positives with the program ROC_AUC [41]. A ROC value of 0.5 means a model accuracy of 50% in predicting positives and is no better than the random assignment of positive or negative status. A ROC value of 1.0 shows the model accurately classified 100% of points. If the area under the resulting curve (AUC) exceeds the critical value of 0.7, the model has high predictive power [42]. To evaluate accuracy, the model was applied to the pool of testing points. A ROC curve was calculated for these points using their predicted ROI value against their experimental AIV-positive status.

## Results

### Important predictor variables

**Figure 2:**
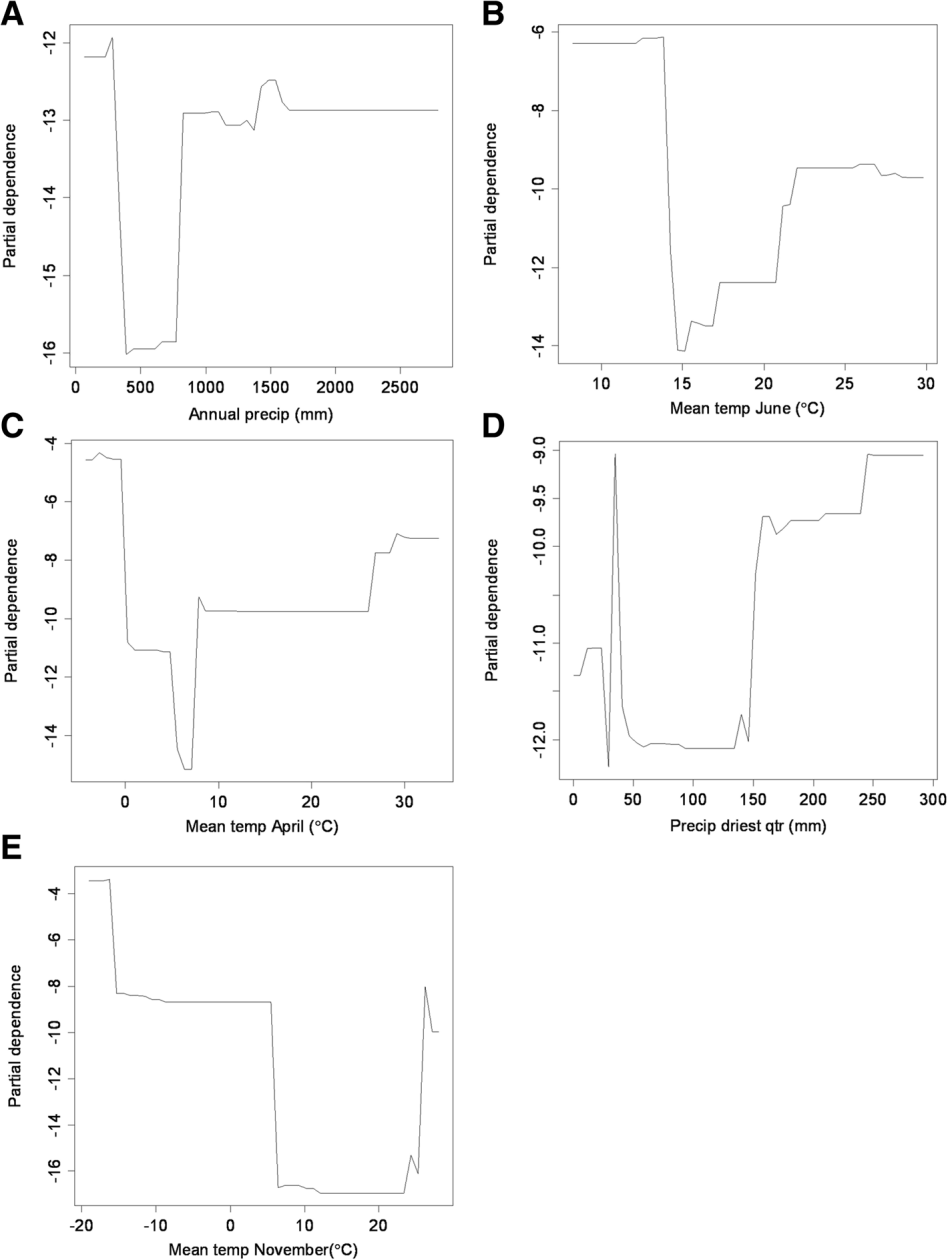
**Partial dependence plots for important variables.** Partial dependence plots for the variables with the five highest variable importance scores as calculated by Random Forests in the accuracy of the predictive model of avian influenza in wild birds (**A**-**E**). Plots show the partial dependence of a high Relative Occurrence Index value for avian influenza on each predictor variable.

Annual precipitation, mean temperature in June, and mean temperature in April were the most important predictor variables with VIS of 100, 85.2, and 76.1, respectively. Predictor variables with VIS above 50 were split almost equally between precipitation measurements and the mean temperatures in November, the driest quarter (3 month period), and annual mean temperature (Table 1). In the density plots (Figure 1a-e) the relative frequency of sampling was approximated by the density of the AIV-negative group of samples (represented by the solid black line); the range of values over which sampling occurred was inferred from the AIV-negative group. In general, the lack of perfect correspondence between AIV-positive (dotted red line) and AI–negative groups showed that there were unequal densities of AIV-positivity across the sampling range. Thus AIV-positive samples did not occur at the same relative frequency as sampling effort. The ranges where the density of AIV-positive samples exceeded those of AIV-negative samples imply conditions correlated with AIV-positivity. In the case of annual precipitation (Figure 1a), moderate (1400 mm) and very low (~0 mm) values were correlated with AIV-positivity. The partial dependence of AIV-positivity on annual precipitation exhibited a similar trend: very high dependence at 0 mm, a trough, and then moderately high dependence at values over 1000 mm (Figure 2a). These patterns imply that areas of low annual precipitation are most correlated with AIV-positivity, although areas of relatively high annual precipitation show some correlation as well. Areas of very low and high mean temperatures in June and April were correlated with AIV-positivity (Figures 1b,c), while areas of moderate temperature were not. June and April displayed similar patterns with a strong peak at the high range of sampling (~28°C and 30°C, respectively) and at the lowest ranges (~10°C and 0°C, respectively). Examination of partial dependence revealed that AIV-positivity was high at the lowest temperatures, dropped sharply at moderate temperatures, and gradually increased at the higher end of the range (Figures 2b,c). Thus areas with low temperatures in June and April were correlated with AIV-positive samples. Precipitation of the driest quarter displayed one peak at 50 mm where AIV-positives had a higher density than AIV-negatives (Figure 1d). However, while the partial dependence was high at this value, it appears as a lone spike in an area of low partial dependence. Partial dependence on precipitation increases above 150 mm and reaches high levels above 250 mm (Figure 2d). While the highest density of AIV-positives occurred at relatively low annual precipitation, partial dependence was highest at the highest range during the driest quarter, which may reflect a low seasonality or variation in rainfall during the year. Mean temperature in November was correlated with AIV-positivity at low and high values (Figures 1e and 2e). Highest partial dependence occurred at the lowest ranges (< −20°C). Based on the important predictor variables, the niche of AIV-positive samples in this study was described as regions of low annual rainfall and low temperatures. There appears to be a secondary niche that described regions of high precipitation and higher temperatures.

### Ecological niche model

**Figure 3:**
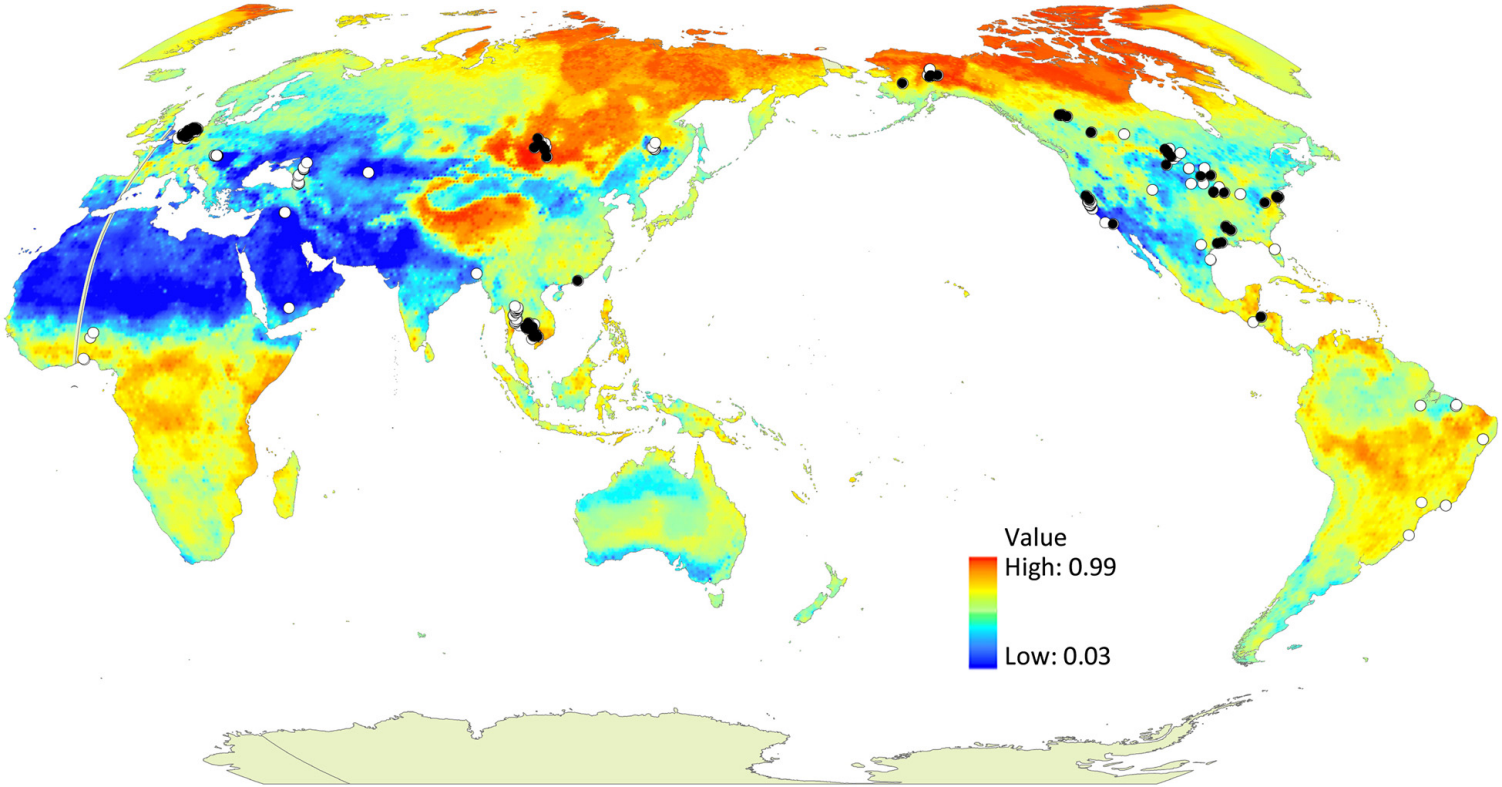
**Global map of the predicted relative occurrence of avian influenza virus (AIV) in wild birds.** The predictive model was constructed using the Random Forests algorithm on 41 predictor variables. The dots on the map represent all samples in both the testing and training databases. Locations where one or more AIV-positive samples were collected are shown as black dots; locations where no positive samples were collected are marked with white. A single dot may represent multiple samples taken at that location. This map is presented in Robinson (sphere) projection, central meridian 145°.

Random Forests produced a robust ecological niche model for AIV in wild birds and identified important predictor variables. The model had an ROC/AUC of 0.79 on the training points and 0.76 on the testing points, lending high confidence to its prediction of the relative occurrence of AIV in wild birds on a global scale. The negative control test was performed by calculating AUC for the training subset against Annual Precipitation (the highest scoring predictor, AUC = 0.59), Mean Temperature in May (the lowest scoring predictor, AUC = 0.47), and Annual Mean Temperature (AUC = 0.47). Although Annual Precipitation received a higher AUC value than the other predictors, this AUC still did not reach the acceptability threshold of 0.7, demonstrating that individual variables were poor predictors of AIV. Northern areas had the highest values of Relative Occurrence Index and temperate regions had the lowest (Figure 3). Interestingly, an equatorial band of relatively high predicted occurrence was observed, which may reflect regions characterized by the secondary niche.

## Discussion

While much of AIV modeling has focused on low-latitude regions and HPAI H5N1, we demonstrated that northern regions are important when all strains of AIV and wild reservoir species are taken into account. By creating a global-scale model, we identified important areas of high predicted occurrence that were missed by AIV models for temperate and sub-tropical regions. Small, local models are vital developing strategies for managing acute outbreaks of specific diseases. However, a global scale perspective is necessary for AIV because, unlike other diseases, is carried by a host that is capable of migrating long distances and potentially infecting others along its path. Furthermore, a model that excludes wild birds, which are the natural reservoir for the virus, neglects the source of gene segments for future infections and potential pandemic strains.

Our model represents the first global-scale predictive map of AIV in wild birds. Using available global AIV data, we identified northern areas as having the highest relative predicted risk of outbreak. Important predictor variables included low temperatures and low annual precipitation. Cold winters and low rainfall may represent continental climates at high latitudes. Areas with these types of climatic conditions include landscapes in Siberia, the Russian Far East, Mongolia, and northern Canada, all of which had high indices of relative occurrence of AIV. Similar conditions at lower latitudes may be created by high elevation, such as the climate of the Tibetan Plateau, which also had a high score. The partial dependence of AIV-positivity on rainfall was bimodal and peaked at very low and high values. This apparently contradictory finding that extremes in rainfall were correlated with AIV-positivity may be explained through laboratory studies of transmission and persistence of the virus. Aerial, non-contact transmission of influenza between guinea pigs was most efficient below 35% relative humidity [43]; thus, we expect dry climate to be conducive to the aerial spread of virus. At low relative humidity and temperature (~6°C, < 46% rh), virus persisted over two weeks on metal, glass, and in soil [44]. Wet conditions and low temperatures were also conducive to viral persistence: the virus remains viable nearly ten times longer in 17°C water than 28°C water [45]. At low temperatures and high relative humidity (~7°C, ~88% rh), the virus persisted over two weeks in chicken feces [44]. Low temperature is the common factor in these studies. While low relative humidity contributes to transmission and persistence on smooth surfaces, the virus also remains viable in water and damp materials such as bird feces. As the virus is transmitted efficiently in water, either through the fecal-oral route [46] or via tracheal shedding [47], dabbling ducks (such as *Anatidae*) in cool northern regions may be at increased risk of contracting AIV from the environment.

Our findings differed from other AIV models in the importance and range of anthropogenic variables. In our model, anthropogenic factors were represented by human population density as well as the Human Influence Index and the Human Footprint Index [32], which are indices calculated based on human population density, land transformation, transportation infrastructure, and electrical power infrastructure. All the anthropogenic variables received very low VIS with human population density scoring the highest at 29.3. Previous models identified high human population density and high farming intensity (especially rice cropping and aquaculture) as important predictors [19,20,48]. The niche they described is characterized as having a high human population, high level of anthropogenic disturbance, and the high annual temperature and humidity of the sub-tropical climates for which the models were designed (i.e. Bangladesh, Vietnam, and Thailand). However, these studies were specific to HPAI H5N1 in poultry. While the one North American model in wild birds identified low minimum temperatures, with which our model was consistent, they also identified the amount of cropland as an important factor [22]. In general, our model did not predict high occurrence of AIV in the continental United States when compared to northern regions, which have not been modeled previously.

Our model demonstrated a novel use of surveillance data that goes beyond the yearly reporting of infected species and viral subtypes isolated. The application of environmental data, GIS, and machine-learning extends the usefulness of surveillance results. However, the prediction of relative occurrence presented here is not a final, definitive map of avian influenza in wild birds, but rather an initial attempt that demonstrates that a useful signal can be gleaned from the noise found in a global dataset. Indeed, it serves to highlight shortcomings in available data. In particular, nearly all data were collected in the Northern Hemisphere. In addition, this Northern Hemispheric niche could then be tested on southward-migrating birds to see if the same predictions are applicable. A predominance of *Anatidae* could create a spatial bias for northern regions and a temporal bias for summer months if most sampling is carried out during summer breeding season at high latitudes. However, if one uses the mean temperature in November as a proxy for latitude, there appears instead to be a strong temperate bias in collection with AIV-positive peaks occurring to either side. The bifurcate niche evident here is an interesting topic for future analysis. The mechanisms responsible for this niche require further investigation in order to clarify how the important bioclimatic variables contribute to AIV-positivity.

While ongoing surveillance is important to understanding the dynamics of AIV, efforts should include wilderness areas, such as Siberia, that have received less attention. Models such as this one could receive additional fine-tuning if these results were to guide future sampling efforts in regions of high predicted occurrence, much of which remains unsampled. As both AIV-positive and AIV-negative data are incorporated into this model, all results from prediction-guided sampling strengthen the prediction, even if only a small percentage of AIV-positive samples are isolated. Given the sheer quantity of data collected by long term surveillance efforts, an unprecedented opportunity exists to produce future models of greater accuracy. If data were curated and publically available, models could be treated as transparent, replicable science experiments. Improved global scale models could not only increase the understanding of viral ecology, but also serve to guide the management of influenza risk policy for the benefit of public health on a global scale. A global model of AIV must be a collaborative effort and we hope this initial attempt encourages greater cooperation and data-sharing among members of the AIV research community.

## Competing interests

The authors declare that they have no competing interests.

## Authors’ contributions

KH carried out the research and the manuscript preparation. ML provided expertise in data analysis. FH was involved in critical review of the manuscript and gave final approval of the version to be published. All authors read and approved the final manuscript.
